# Artificial intelligence literacy among nursing students and its association with learning engagement

**DOI:** 10.3389/fpubh.2026.1928543

**Published:** 2026-08-14

**Authors:** Min Li, Yue Cao, Ruilin Zhang, Jingquan Gao, Yuqian Ma, Xuefen Lan

**Affiliations:** Department of Nursing Sciences, Lishui University, Lishui, China

**Keywords:** artificial intelligence literacy, latent profile analysis, learning engagement, technology acceptance model, undergraduate nursing students

## Abstract

**Background:**

Current research does not examine how distinct AI literacy profiles are differentially associated with learning engagement, thereby impeding the development of stratified and precise training plans for nursing students.

**Objective:**

To identify latent profiles of artificial intelligence literacy among undergraduate nursing students, characterize their distributional features, and examine the relationship between distinct AI literacy profiles and learning engagement.

**Methods:**

The study included 479 Chinese undergraduate nursing students who finished the Utrecht Work Engagement Scale-Student Version and the Artificial Intelligence Literacy Scale. Latent profile analysis was conducted using item-level AI literacy scores as manifest indicators.

**Results:**

Three distinct profiles of AI literacy were identified: low literacy—ethically cautious, medium literacy—balanced development, and high literacy—fully mature. Non-parametric test results demonstrated significant differences in learning engagement and its dimensions across the three AI literacy profiles. After controlling for relevant confounding factors in multilevel linear regression analyses, AI literacy profile remained significantly associated with learning engagement, accounting for an additional 31.2% of the variance. Students in the medium and high AI literacy groups demonstrated significantly higher levels of learning engagement compared to those in the low literacy group.

**Conclusion:**

Undergraduate nursing students’ AI literacy is heterogeneous and markedly related to learning engagement. These findings provide valuable insights for improving student engagement in AI-supported learning environments.

## 1 Introduction

Amid the rapid advancement of intelligent healthcare systems worldwide, artificial intelligence (AI) has been increasingly integrated into nursing practice (1). For nursing students, AI literacy has become a crucial skill. It includes the capacity to recognize, comprehend, apply, assess, and consider AI technology in both educational and therapeutic settings (2), and is increasingly regarded as a key indicator of professional readiness in nursing education (1). In recent years, a growing body of research has focused on the conceptualization, current status, and determinants of AI literacy among nursing students, providing valuable insights into its developmental characteristics and educational implications (3). In Australia, Ghimire and Qiu (4) emphasized the role of personalized, student-driven AI interaction in fostering autonomous learning engagement. Similarly, in the United States, Khatun et al. (5) reported widespread yet unstructured generative AI use among nursing students, highlighting a critical gap between usage frequency and formal ethical guidance. In Europe, Dissanayake et al. (6) synthesized evidence from multiple countries and called for standardized AI competency frameworks across nursing curricula. Studies have shown that substantial individual differences exist among Chinese nursing students in terms of AI-related knowledge acquisition, technical application, and ethical reflection (7). Despite these advances in AI-related research, most existing studies still adopt a variable-centered approach. To date, most studies have conceptualized AI literacy as a continuous variable and have predominantly concentrated on influence of external factors, including demographic characteristics, curriculum design, and technological exposure, on overall literacy levels (3). Although these variable-centered approaches have contributed to identifying key determinants, they tend to overlook the multidimensional and heterogeneous nature of AI literacy at the individual level, particularly across domains such as knowledge, skills, evaluation, and ethics (7). As a result, they offer limited insight into the underlying structural patterns and developmental profiles of AI literacy among students (3). Latent Profile Analysis (LPA) provides a robust framework for identifying unobserved subgroups within a given population by utilizing multiple dimensional indicators (8). By capturing heterogeneity in competency structures, LPA may enhance our understanding of how AI literacy manifests across different student groups. Traditional regression analysis treats AI literacy as a single continuous composite variable, revealing only average associations across the entire sample and thereby obscuring heterogeneous subgroups with distinct AI literacy profiles. In contrast, LPA classifies participants into different latent subtypes based on their multidimensional performance in AI literacy, thus identifying unique groups that traditional aggregate regression methods fail to detect. Emerging studies have applied LPA to categorize students’ AI literacy levels and have demonstrated the predictive roles of demographic characteristics and training experiences in determining profile membership (9). Nonetheless, there remains a paucity of empirical evidence regarding how different AI literacy profiles relate to core academic outcomes and learning processes, especially learning engagement, a key construct reflecting students’ vigor, dedication, and immersion in educational activities (9). This gap results in an incomplete research framework linking “antecedents of literacy-literacy profiles-academic outcomes,” thereby constraining the development of theoretically grounded and targeted educational interventions. Learning engagement, conceptualized within the framework of positive psychology by Schaufeli (10), represents a multidimensional construct comprising the components of vigor, dedication, and absorption. It reflects students’ emotional experience, behavioral participation, and cognitive investment in the learning process (11). As a critical mediating variable in predicting academic achievement, professional identity, and competency development, learning engagement serves not only as a key indicator of the quality of professional learning among undergraduate nursing students but also as a key marker of their developmental potential (12–14). Despite its importance, empirical research investigating the relationship between AI literacy and learning engagement remains scarce (15). Based on social cognitive theory, individuals’ technological competence and cognitive capabilities directly shape their self-efficacy and motivational processes in learning (16). Nursing undergraduates with a higher level of AI literacy can more efficiently utilize intelligent tools to integrate learning resources, solve professional nursing problems, and construct knowledge systems, thereby enhancing their learning vitality and concentration, and maintaining a high level of learning engagement (16). Students with limited AI literacy may encounter barriers in technology use, leading to frustration, reduced self-efficacy, and ultimately diminished learning engagement (1). However, existing studies have yet to elucidate heterogeneous relationships meaningful individual differences, thereby limiting the precision and effectiveness of educational interventions.

Accordingly, this study adopts a person-centered methodology to delineate latent profiles of AI literacy among nursing students using LPA. It seeks to offer empirical evidence to guide the establishment of stratified and targeted educational strategies for AI literacy.

## 2 Methods

### 2.1 Research design and participants

This multicenter cross-sectional study was conducted from February to March 2026. A convenience sampling approach was utilized to enroll undergraduate nursing students from three universities located in Zhejiang Province, China. The selected institutions are geographically distributed across the southern, eastern, and northern regions of the province, providing a degree of regional representativeness. The inclusion criteria comprised: (1) Full-time undergraduate nursing students; (2) Those who have given informed consent and voluntarily participated. Exclusion criteria included: (1) Those with mental or psychological disorders; (2) Individuals who have been absent from their studies for a duration exceeding 1 month due to medical or personal leave.

It is generally recommended that each latent class include at least 30 participants, and that the total sample size be 10–40 times the number of observed indicators to ensure robust classification (17). Given that the study encompassed 12 indicators, a minimum sample size of 360 was required based on a 30-fold estimation. Allowing for an anticipated 10% rate of invalid responses, the target sample size was adjusted to 400. Ultimately, 479 valid questionnaires were obtained.

### 2.2 Instruments

#### 2.2.1 General information questionnaire

A self-administered questionnaire was constructed following a comprehensive review of pertinent literature (18). It comprised seven items capturing participants’ demographic and academic characteristics: gender, academic year, monthly living expenses, motivations for selecting nursing as a major, level of familiarity with AI applications in nursing, frequency of AI tool usage, and perceived impact of AI on nursing education.

#### 2.2.2 Artificial intelligence literacy scale

AI literacy was assessed using the AILS, developed by Wang and colleagues (2), in accordance with the AI Literacy Framework. The scale comprises four dimensions: awareness, usage, evaluation, and ethics, each represented by three items (12 items in total). Participants responded on a 7-point Likert scale, with options ranging from 1 (“strongly disagree”) to 7 (“strongly agree”). Items 2, 5, and 11 were reverse-coded. Higher scores indicate greater AI literacy. The Cronbach’s *α* of the measurement scale was 0.92, and the content validity index (CVI) ranged from 0.80 to 0.85 (2). In this study, the Cronbach’s α was 0.85.

#### 2.2.3 Utrecht work engagement scale-student

Learning engagement was assessed using the UWES-S, adapted from the original scale developed by Schaufeli et al. (10) and subsequently validated in Chinese by Fang et al. (11). The scale includes three dimensions—vigor, dedication, and absorption, comprising 17 items. Respondents rated each item on a 7-point Likert scale ranging from 1 (“never”) to 7 (“always/every day”), with higher scores reflecting greater levels of learning engagement. The scale exhibited excellent reliability, with a Cronbach’s alpha of 0.95. In this study, the Cronbach’s *α* was 0.87.

### 2.3 Ethics

This study adhered to the ethical principles of the Declaration of Helsinki and was approved by the Ethics Committee (Approval Number: RTYX2026003). All scales used in this study were authorized by their developers, and all research subjects provided signed informed consent forms.

### 2.4 Pilot study

A pilot study was conducted prior to the main survey to evaluate the questionnaire’s clarity, comprehensibility, feasibility, and respondent burden. A convenience sample of 50 undergraduate nursing students was recruited. To preserve data integrity, these participants were excluded from the final analytical sample. The results indicated that no structural or conceptual modifications were needed. Cronbach’s *α* coefficients were 0.91 for the AI Literacy Scale and 0.92 for the Utrecht Work Engagement Scale– Student, indicating strong internal consistency. The mean completion time was 12 min, confirming acceptable respondent burden and procedural efficiency.

### 2.5 Data collection

All measurement instruments employed in this study were administered with permission from their original developers. Data were gathered via an online survey hosted on the Wenjuanxing platform. Following approval from the nursing program directors at the three participating institutions, a QR code directing to the electronic questionnaire was disseminated to student class groups from February to March 2026. The first page of the questionnaire provided standardized instructions outlining the study purpose, completion requirements, and key considerations. Participation was voluntary, and students were required to give informed consent before proceeding to the survey items. Several items were reverse-coded to reduce response pattern bias. In addition, anonymity was emphasized, instructions were standardized, and no leading cues were provided during data collection. To maintain data quality, submissions were limited to one per device, and all questionnaire items were designated as mandatory. Data entry was independently verified by two researchers. A total of 520 questionnaires were initially collected. Forty-one questionnaires were removed due to patterned responses and abnormal completion times (either too short [<80 s] or too long [>30 min]). Ultimately, 479 valid questionnaires were retained, resulting in an effective response rate of 92.1%.

### 2.6 Data analysis

Data analysis was performed utilizing SPSS v28.0 and Mplus v8.3. Continuous variables exhibiting normal distributions were summarized as means accompanied by standard deviations (mean ± SD), while categorical variables presented in terms of frequencies and percentages. LPA was subsequently conducted in Mplus. The following criteria were applied: (1) Akaike Information Criterion (AIC), Bayesian Information Criterion (BIC), and adjusted BIC (aBIC), with lower values indicating better model fit; and entropy, with values approaching 1 reflecting greater classification accuracy. (2) The Lo–Mendell–Rubin likelihood ratio (LMR) test and the bootstrap likelihood ratio test (BLRT), which compare k-class and (k-1)-class models. A *p*-value < 0.05 indicates that the k-class model provides a significantly better fit than the (k-1)-class model (19). Following profile classification, and based on the model fit results described above, chi-square tests (Pearson’s chi-square) were conducted to compare the general characteristics of undergraduate nursing students with different AI literacy profiles. The Kruskal-Wallis H test was used to examine differences in learning engagement and its dimension scores among the groups. In cases where the overall test yielded statistically significant results, subsequent *post hoc* pairwise comparisons were conducted using the Bonferroni correction.

## 3 Results

### 3.1 Assessment of common method variance

Harman’s single-factor test was conducted to assess the potential for common method variance. The results indicated that six factors with eigenvalues greater than 1 were extracted. The first (largest) factor accounted for 29.64% of the total variance, which is below the recommended threshold of 40% (20). These findings suggest that common method bias was not a significant concern in the present study.

### 3.2 Latent profile analysis of AI literacy

**Figure 1:**
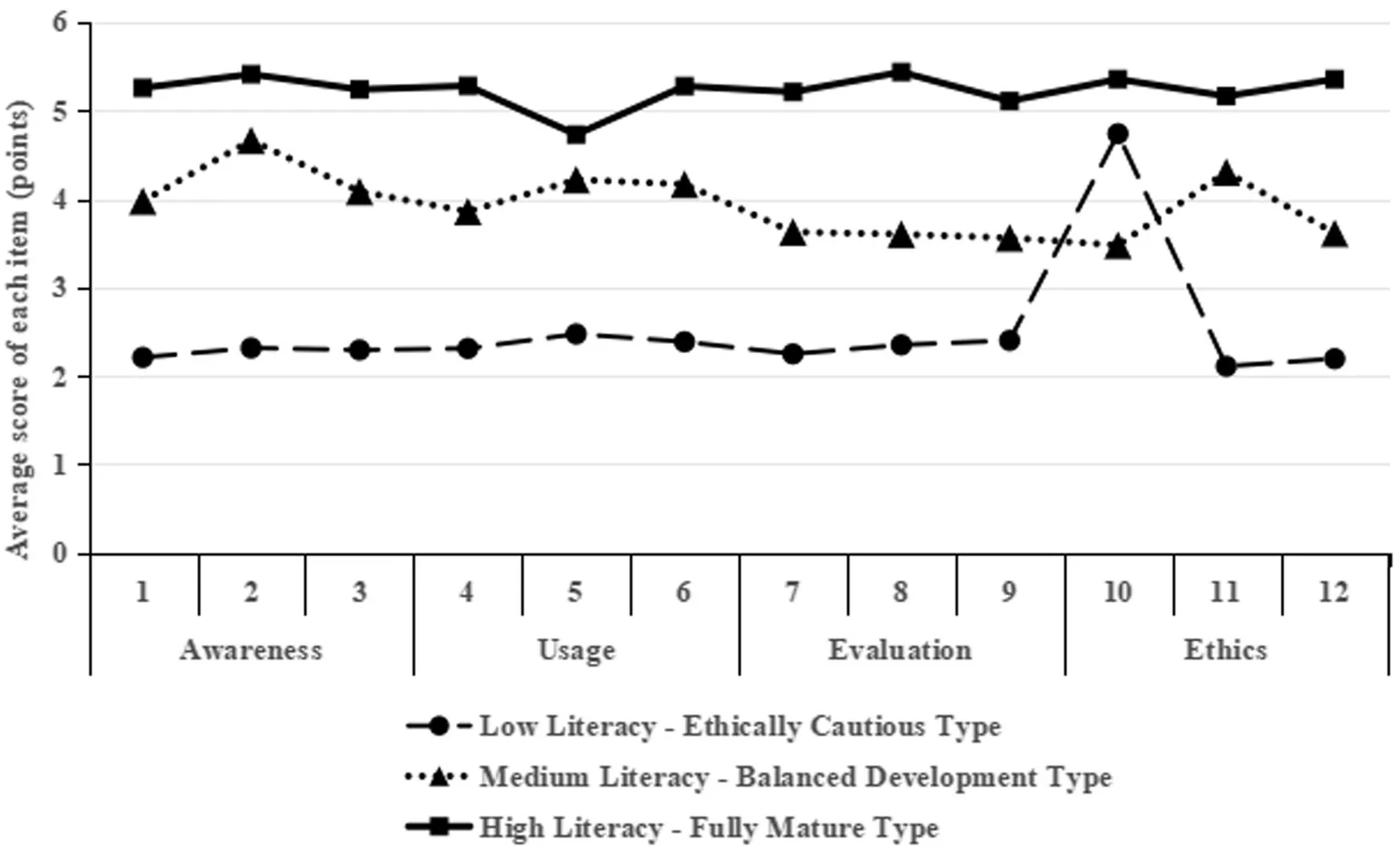
Profile plot of AI literacy latent classes (latent profile analysis based on four dimensions).

LPA was performed using the 12 item-level indicators of AI literacy from 479 students. Models specifying one to four latent classes were estimated sequentially, and model fit indices are summarized in Table 1. Parameters were estimated using maximum likelihood estimation with robust standard errors (MLR). Missing data were handled using full information maximum likelihood (FIML) under the missing-at-random assumption. To reduce the risk of converging on local maxima, 500 random sets of starting values were used in the initial stage, followed by 100 final stage optimizations. As the number of classes increased, the values of the AIC, BIC, and adjusted BIC decreased progressively, indicating improved model fit (19). For the four-class solution, the LMR test was not statistically significant, suggesting no meaningful improvement over the three-class model. Although the two-class model exhibited the highest entropy value, the three-class model demonstrated superior interpretability and practical relevance. In addition, the average posterior probabilities for class membership in the three-class model were 98.0, 95.4, and 93.9%, respectively, indicating high classification accuracy. Based on variations in relative levels and structural attributes across the three dimensions, the subclasses were designated as Low Literacy–Ethically Cautious, Moderate Literacy–Balanced Development, and High Literacy–Fully Mature, accounting for 29.0, 56.0, and 15.0% of the total sample (Figure 1).

**Table 1:** Comparison of model fit indices for latent profiles of AI literacy.

Model	L	AIC	BIC	aBIC	Entropy	LMR	BLRT	Category probability
1	−10956.25	21960.49	22060.62	21984.44	–	–	–	1
2	−10282.50	20639.00	20793.36	20675.92	0.931	<0.001	<0.001	0.31/0.69
3	−10060.91	20221.82	20430.41	20271.72	0.907	0.017	<0.001	0.29/0.56/0.15
4	−9966.51	20059.02	20321.83	20121.88	0.858	0.173	<0.001	0.29/0.31/0.26/0.13

AIC, Akaike Information Criterion; BIC, Bayesian Information Criterion; aBIC, Sample-Adjusted Bayesian Information Criterion; LMR, Lo–Mendell–Rubin Likelihood Ratio Test; BLRT, Bootstrap Likelihood Ratio Test.

### 3.3 Differences in participant characteristics across latent profiles

Significant differences in AI literacy latent subtypes membership were observed across several variables, including academic year, level of familiarity with AI applications in nursing, frequency of AI tool usage, and perceived impact of AI on professional learning (*p* < 0.05). No statistically significant differences were detected for other demographic variables (Table 2).

**Table 2:** Comparison of demographic characteristics across latent profiles of AI literacy (*n* = 479).

Variable category	Item	Number	R1 (*n* = 139)	R2 (*n* = 268)	R3 (*n* = 72)	χ^2^	*P*
Gender	Male	96 (20.0)	31 (22.3)	56 (20.9)	9 (12.5)	3.12	0.210
	Female	383 (80.0)	108 (77.7)	212 (79.1)	63 (87.5)		
Grade	Freshman year	110 (23.0)	65 (46.8)	31 (11.6)	14 (19.4)	199.85	<0.001
	Sophomore year	98 (20.5)	63 (45.3)	29 (10.8)	6 (8.3)		
	Junior year	145 (30.3)	6 (4.3)	103 (38.4)	36 (50.0)		
	Senior year	126 (26.3)	5 (3.6)	105 (39.2)	16 (22.2)		
Living expenses (per month)	≤2000	132 (27.6)	41 (29.5)	69 (25.7)	22 (30.6)	4.91	0.297
	2001 ~ 3,000	202 (42.2)	65 (46.8)	108 (40.3)	29 (40.3)		
	≥3,000	145 (30.3)	33 (23.7)	91 (34.0)	21 (29.2)		
Primary reasons for choosing the nursing profession	Personal interests and career aspirations	165 (34.4)	46 (33.1)	97 (36.2)	22 (30.6)	3.61	0.729
	Advice or influence from family members	113 (23.6)	32 (23.0)	64 (23.9)	17 (23.6)		
	Good employment prospects	146 (30.5)	47 (33.8)	73 (27.2)	26 (36.1)		
	Adjustment	55 (11.5)	14 (10.1)	34 (12.7)	7 (9.7)		
Familiarity with AI applications	Poor understanding	149 (31.1)	73 (52.5)	49 (18.3)	27 (37.5)	53.01	<0.001
	Moderate understanding	172 (35.9)	30 (21.6)	119 (44.4)	23 (31.9)		
	Good understanding	158 (33.0)	36 (25.9)	100 (37.3)	22 (30.6)		
Frequency of AI tool usage	Several times per week	164 (34.2)	39 (28.1)	94 (35.1)	31 (43.1)	12.07	0.017
	Several times per month	174 (36.3)	51 (36.7)	92 (34.3)	31 (43.1)		
	Several times per semester	141 (29.4)	49 (35.3)	82 (30.6)	10 (13.9)		
Evaluation of AI’s impact on nursing	Slightly helpful	158 (33.0)	71 (51.1)	76 (28.4)	11 (15.3)	34.26	<0.001
	Moderately helpful	162 (33.8)	34 (24.5)	100 (37.3)	28 (38.9)		
	Highly helpful	159 (33.2)	34 (24.5)	92 (34.3)	33 (45.8)		

R1, Low Literacy – Ethically Cautious Type; R2, Medium Literacy – Balanced Development Type; R3, High Literacy – Fully Mature Type; χ2, Chi-square statistic.

### 3.4 Factors associated with learning engagement among undergraduate nursing students

#### 3.4.1 Univariate analysis

Univariate analyses revealed significant differences in learning engagement across several demographic and AI literacy latent subtypes. Specifically, learning engagement scores varied significantly by academic year (H = 56.51, *p* < 0.001), level of understanding of AI applications in nursing (H = 26.64, *p* < 0.001), frequency of AI tool usage (H = 10.60, *p* = 0.005), and perceived impact of AI on professional learning (H = 45.27, *p* < 0.001). Furthermore, significant differences were observed in overall learning engagement as well as its three dimensions, vigor, dedication, and absorption, across the identified AI literacy latent subtypes (Table 3).

**Table 3:** Differences in AI literacy latent subtypes across learning engagement and its dimensions (*n* = 479).

[M (Q1, Q3)]					
Variable	R1 (*n* = 139)	R2 (*n* = 268)	R3 (*n* = 72)	H	Post hoc comparisons
Study engagement	53 (47, 61)	66 (60, 72)	90 (71.25, 95.75)	208.99***	R1 < R2 < R3
Vigor dimension	18 (15, 20)	23 (20, 27)	32 (26, 27)	170.18***	R1 < R2 < R3
Dedication dimension	17 (13, 20)	19 (17, 22.75)	26 (22, 28)	105.02***	R1 < R2 < R3
Absorption dimension	18 (15, 22)	23 (19, 26.75)	23 (19, 26.75)	151.19***	R1 < R2 < R3

R1, Low Literacy – Ethically Cautious Type; R2, Medium Literacy – Balanced Development Type; R3, High Literacy – Fully Mature Type; H, Kruskal-Wallis H statistic. *** *p* < 0.001.

#### 3.4.2 Multivariate analysis

Hierarchical multiple linear regression analysis was conducted to investigate the independent relationship between AI literacy latent subtypes and learning engagement. Learning engagement was entered as the dependent variable. Based on the univariate findings, academic year, level of familiarity with AI applications in nursing, frequency of AI tool use, and perceived impact of AI on professional learning were included as control variables in Step 1 (Supplementary material 1). AI literacy latent subtypes were subsequently included in Step 2. All variance inflation factor (VIF) values were below 10, indicating the absence of multicollinearity (Supplementary material 2). After controlling for covariates, AI literacy latent subtypes remained significantly associated with learning engagement. Compared with the low literacy-ethically cautious group, both the moderate literacy-balanced development group and the high literacy-fully mature group demonstrated remarkably higher levels of learning engagement, accounting for an additional 17.1% of the variance (*Δ R^2^* = 0.312, *p* < 0.001). The comprehensive regression results are presented in Table 4.

**Table 4:** Hierarchical multiple linear regression analysis of learning engagement (*n* = 479).

Independent variable	Model 1			Model 2		
	*β*	SE	*β*’	*β*	SE	*β*’
Constant term	53.607	1.754		50.709	1.397	
Sophomore year	−3.667	1.788	−0.104*	−2.282	1.397	−0.064
Junior year	8.154	1.646	0.262***	−0.538	1.466	−0.017
Senior year	3.681	1.706	0.113***	−2.829	1.516	−0.087
Several times per week	3.882	1.518	0.129*	0.872	1.2	0.029
Several times per month	3.623	1.502	0.122*	0.753	1.184	0.025
Moderately helpful	6.245	1.494	0.207***	3.093	1.179	0.102**
Highly helpful	7.819	1.52	0.258***	3.593	1.21	0.118**
Moderate understanding	2.586	1.52	0.087	2.664	1.233	0.089*
Medium literacy – balanced development type				12.185	1.408	0.423***
High literacy – fully mature type				29.688	1.72	0.742***
*F*	13.757***			46.192***		
R2	0.209			0.521		
Adjusted R2	0.194			0.510		
R2 Change	0.209			0.312		

* *P* < 0.05; ** *P* < 0.01; *** *P* < 0.001.

## 4 Discussion

### 4.1 Latent profile characteristics of AI literacy

This research employed LPA to delineate three distinct profiles of AI literacy among undergraduate nursing students. These findings differ from those reported by Zhang (21), who identified four AI literacy profiles among a general college student population in China. This variation may be ascribed to disparities in study populations and measurement approaches. Specifically, the current study focuses on undergraduate nursing students, whose AI literacy is shaped by discipline-specific educational requirements and clinical practice settings. In contrast, Zhang (21) examined a more heterogeneous sample of college students and employed a distinct set of measurement dimensions and indicators, which may have contributed to the identification of a more granular classification structure. Despite these differences, both lines of research consistently highlight substantial heterogeneity in AI literacy among student populations.

The present findings indicate that 29.0% of undergraduate nursing students were categorized within the low literacy-ethically cautious profile. This group demonstrated consistently low scores across the domains of knowledge, technical application, and evaluative judgment, while exhibiting comparatively higher levels of ethical awareness. Nevertheless, their heightened ethical awareness may serve as a protective factor, promoting cautious and responsible use of AI technologies (7). The largest proportion of participants (56.0%) belonged to the moderate literacy-balanced development profile. Students in this group exhibited mid-range scores across all four dimensions, indicating relatively even but non-specialized development. This distribution suggests that the majority of undergraduate nursing students possess foundational knowledge and basic operational skills related to AI, yet have not achieved deeper integration or higher-order application. This finding aligns with the current status of AI education in nursing, which remains largely oriented toward introductory knowledge dissemination, with limited emphasis on systematic curriculum design and integration with clinical practice (1, 22, 23). These patterns differ markedly from those reported in Western cohorts. For instance, Australian nursing students tend to exhibit a highly polarized profile characterized by either strong self-directed AI exploration or active resistance (4), whereas American students demonstrate a “High Usage-Low Policy Awareness” pattern (5). In contrast, our findings reveal a more evenly distributed and moderately engaged profile structure, likely reflecting the influence of China’s collectivist educational culture, where AI tools are primarily integrated as instructor-guided supplements rather than as platforms for independent inquiry. In contrast, 15.0% of students were classified as the high literacy-fully mature profile, characterized by consistently high scores across all dimensions. The relatively low proportion suggests that undergraduate nursing students are still in the early stages of systematically integrating artificial intelligence knowledge, application skills, and ethical reasoning (24). At the same time, existing educational models, largely focused on basic AI knowledge and operational skills, may lack opportunities for research engagement, clinical application, and interdisciplinary collaboration, thereby limiting the continued advancement of students with higher levels of AI literacy (24).

In summary, the findings highlight notable limitations in current nursing education regarding the cultivation of AI literacy. To address these challenges, AI education in nursing should transition from a generalized training approach to a precision-oriented empowerment paradigm grounded in competency profiling (25). Specifically, for students in the low literacy-ethically cautious profile, instructional efforts should utilize their relative strength in ethical sensitivity while enhancing motivation through contextualized, case-based learning. This should be complemented by structured, low-risk simulation training to build technical self-efficacy and reduce technology-related anxiety. For the moderate literacy-balanced development group, emphasis should be placed on strengthening higher-order competencies, particularly in critical evaluation and ethical reasoning. Students should be guided to engage in reflective practice, focusing on issues such as algorithmic transparency, data representativeness, and clinical applicability. For the high literacy-fully mature group, educational environments should facilitate participation in advanced, real-world “AI + nursing” initiatives, such as intelligent chronic disease management and the development of clinical decision support systems, thereby promoting knowledge integration and innovation in complex, practice-based settings.

### 4.2 Factors associated with latent subtypes of AI literacy

The present study identified several key factors associated with AI literacy latent subtypes, including academic year, level of familiarity with AI applications, frequency of AI tool use, and perceived impact of AI on professional learning (*p* < 0.01). Notably, students in the moderate and high literacy profiles were more likely to be senior students, demonstrate greater familiarity with AI applications, report more frequent use of AI tools, and hold more positive perceptions regarding the role of AI in nursing education. These results align with those reported in prior studies (3, 9). Senior students typically possess a more robust disciplinary foundation and greater exposure to clinical and technological settings, which may facilitate the development of higher levels of AI literacy (26). Similarly, students with a deeper understanding of AI are better positioned to integrate theoretical knowledge with practical application, From the perspective of social cognitive theory, leading to greater learning initiative and stronger behavioral intentions to engage with AI technologies (27). Nursing students who recognize the professional value of AI are more likely to develop positive attitudes and intentions toward its use, leading to a reinforcing cycle of value recognition, active engagement, and competency development (28). Building upon prior research, these findings not only confirm the role of key influencing factors but also elucidate the heterogeneous nature of AI literacy among nursing students. Importantly, they provide a foundation for further investigation into the links between distinct AI literacy latent subtypes and learning engagement and support the development of targeted, evidence-based educational interventions.

### 4.3 Factors influencing learning engagement among undergraduate nursing students

The present study identified significant differences in learning engagement across multiple factors, including academic year, frequency of AI tool use, and perceived impact of AI on professional learning (*p* < 0.05). The current findings extend existing evidence by demonstrating that AI-related cognition and usage behaviors are closely associated with students’ engagement in academic activities (29). Further analyses revealed that both overall learning engagement and its three dimensions, vigor, dedication, and absorption, differed significantly across AI literacy profiles (*p* < 0.001). These findings suggest that AI literacy functions as an intrinsic competency that shapes students’ emotional, cognitive, and behavioral involvement in learning processes (29).

Importantly, hierarchical multiple regression analysis confirmed that AI literacy profiles were significantly associated with learning engagement after controlling for relevant covariates, explaining an additional 31.2% of the variance (ΔR^2^ = 0.312, *p* < 0.001). Compared with students in the low literacy-ethically cautious group, those in the moderate literacy-balanced development and high literacy-fully mature groups exhibited significantly higher levels of learning engagement (*p* < 0.001). This observed pattern aligns with previous research and highlights the critical role of AI literacy in academic engagement (15). A plausible explanation is that students possessing greater AI literacy are more adept at incorporating AI tools into their learning processes (24). Through the effective use of intelligent search systems, data analysis tools, and simulation-based training, these students can optimize learning strategies, enhance efficiency, and deepen understanding (24). These findings suggest that improving AI literacy may serve as a viable pathway to enhancing learning engagement among nursing students. AI literacy may enhance learning engagement through the dynamic interplay of personal cognition, behavioral capability, and environmental factors (30). Students with higher AI literacy possess stronger cognitive understanding and operational skills, enabling them to effectively utilize AI tools and generate positive learning experiences. This, in turn, reinforces a virtuous cycle of cognition, behavior, and environment, promoting sustained motivation and concentration (31). Conversely, students in the low literacy-ethically cautious group may experience constrained engagement due to limited technical competence and heightened ethical concerns, which may inhibit their willingness to adopt AI tools and reduce active participation in learning (31). However, it should be noted that the direction of this relationship remains undetermined. Students who are inherently more engaged in learning may have greater exposure to and use of AI-related resources in their daily studies, thereby naturally developing higher AI literacy. In other words, learning engagement might also promote the enhancement of AI literacy. Given that this study is cross-sectional, causality cannot be established, and a bidirectional influence between the two variables is more likely. Future longitudinal research is needed to clarify their temporal sequence and causal pathways. Moreover, emerging evidence suggests that the incorporation of AI into educational practices can significantly enhance higher-order thinking skills and overall competency development (32). In this study, nursing students with medium AI literacy have recognized the potential value of AI in their professional learning (perceived usefulness), but their limited proficiency in applying the technology (insufficient perceived ease of use) may place them in a “wait-and-see” stage regarding technology adoption. Conversely, low-literacy nursing students demonstrate both vague cognition and weak technical skills, leading to lower perceptions of AI’s usefulness and ease of use, weaker technology acceptance, and correspondingly lower levels of study engagement (33). These explanations align with the present findings, which show a clear gradient in learning engagement across the three AI literacy groups (R3 > R2 > R1). Based on these theoretical and empirical insights, nursing education should adopt an AI-integrated, task-driven teaching model closely aligned with clinical scenarios. Such a model should systematically enhance students’ AI evaluation abilities and ethical reasoning, fostering balanced development across technical proficiency, critical judgment, and ethical awareness. By promoting comprehensive AI literacy, this approach can strengthen students’ engagement in learning while cultivating compound nursing professionals equipped with deep learning skills, technical competence, and ethical decision-making capabilities.

## 5 Limitations

This study has several limitations. First, the cross-sectional design captures data at a single time point and cannot establish causal relationships among variables; longitudinal or interventional studies are needed for verification. Second, although this study adopted a multi-center convenience sampling design, all participants were recruited from a single province in China. Consequently, the generalizability of the findings to nursing students in other provinces or regions should be interpreted with caution. Future research should adopt larger-scale, multi-center designs based on probability sampling frameworks, such as stratified random sampling. Third, the dependency on self-reported measures of AI literacy and learning engagement may introduce bias. Subsequent research could incorporate objective metrics, such as AI skill practice assessments or digital learning platform analytics. Fourth, LPA categorization depends on model fit indices and may vary across samples; the stability of these classifications should be tested in independent cohorts. Finally, cultural context may influence findings, as participants were exclusively Chinese nursing undergraduates. It remains to be determined whether these conclusions apply across other cultural or educational settings.

## 6 Conclusion

Although prior regression-based studies have confirmed an overall positive trend, our LPA results further indicate that this relationship is not consistent across all learners. This variability underscores the limitations of universal, standardized interventions for AI literacy development; rather, pedagogical strategies should be differentiated and tailored to students’ empirically identified profiles. Tailored instructional strategies can support the simultaneous enhancement of AI literacy and study engagement, thereby preparing nursing students to meet the evolving demands of “AI + nursing.” Given the study’s regional scope, future research should conduct multi-center, large-sample, and longitudinal studies to examine the sustained impact of AI literacy on learning engagement. Additionally, diverse AI-integrated instructional models, such as AI-assisted clinical decision-making, AI-driven reflective practice, and human-machine collaborative simulations, should be developed and empirically validated to evaluate their differential effects across nursing student subgroups.

## Data Availability

The raw data supporting the conclusions of this article will be made available by the authors, without undue reservation.
