# Supplementary material for: Artificial intelligence literacy among nursing students and its association with learning engagement
Source: Front Public Health. 2026 Aug 14;14:1928543. doi: 10.3389/fpubh.2026.1928543 (PMC13522149; doi:10.3389/fpubh.2026.1928543)
Supplement: Supplementary file 1 [file Table_1.DOCX]

**Supplementary Material 1 Variable coding and measurement**

| Variable | Coding Scheme |
| --- | --- |
| Grade | Freshman year = (0, 0, 0), sophomore year = (1, 0, 0), junior year = (0, 1, 0), senior year = (0, 0, 1) |
| Perceived understanding of AI applications in nursing | Poor understanding = (0, 0), Moderate understanding = (1, 0),Good understanding = (0, 1) |
| Frequency of AI tool utilization | Several times per week = (1, 0), Several times per month = (0, 1), Several times per semester = (0, 0) |
| Evaluation of AI's impact on nursing professional learning | Slightly helpful = (0, 0), Moderately helpful = (1, 0), Highly helpful = (0, 1) |
| Artificial intelligence literacy | Low Literacy - Ethically Cautious Type = (0, 0), Medium Literacy - Balanced Development Type = (1, 0), High Literacy - Fully Mature Type = (0, 1) |
