# Supplementary material for: Artificial intelligence literacy among nursing students and its association with learning engagement
Source: Front Public Health. 2026 Aug 14;14:1928543. doi: 10.3389/fpubh.2026.1928543 (PMC13522149; doi:10.3389/fpubh.2026.1928543)
Supplement: Supplementary file 2 [file Table_2.DOCX]

**Supplementary Material 2 Results of Collinearity Diagnostics for the Regression Model.**

| Items | | VIF | Tolerance |
| --- | --- | --- | --- |
| Grade (Freshman yearr as ref) | Sophomore year | 1.516 | 0.66 |
|  | Junior year | 2.166 | 0.462 |
|  | Senior year | 2.127 | 0.47 |
| Perceived understanding of AI applications in nursing (Poor understanding as ref) | Moderate understanding | 1.669 | 0.599 |
|  | Good understanding | 1.694 | 0.59 |
| Frequency of AI tool utilization(Several times per semester as ref) | Several times per week | 1.547 | 0.646 |
|  | Several times per month | 1.549 | 0.646 |
| Evaluation of AI's impact on nursing professional learning(Slightly helpful as ref) | Moderately helpful | 1.486 | 0.673 |
|  | Highly helpful | 1.55 | 0.645 |
| Artificial intelligence literacy(Low Literacy - Ethically Cautious Type as ref) | Medium Literacy - Balanced Development Type | 2.331 | 0.429 |
|  | High Literacy - Fully Mature Type | 1.804 | 0.554 |
